# ‘My HS is significantly impacted by my cycle’: a cross-sectional study exploring the impact of menstruation and pregnancy on hidradenitis suppurativa

**DOI:** 10.1093/skinhd/vzag087

**Published:** 2026-07-06

**Authors:** Olivia Garrett, Eliza Hutchison, Hannah E Wainman

**Affiliations:** General Medicine, North Bristol NHS Trust, Bristol, UK; Centre for Applied Excellence in Skin & Allergy Research, University of Bristol, Bristol, UK; Dermatology Department, Royal United Hospitals Bath NHS Foundation Trust, Bath, UK; Centre for Applied Excellence in Skin & Allergy Research, University of Bristol, Bristol, UK; Bristol Dermatology Centre, Bristol Royal Infirmary, University Hospitals Bristol and Weston NHS Foundation Trust, Bristol, UK

## Abstract

**Background:**

Hidradenitis suppurativa (HS) is a chronic skin condition that causes painful abscesses, nodules and tunnelling in the skin folds. HS disproportionately affects women, and androgenic hormones are implicated in its pathophysiology.

**Objectives:**

To explore the impact of menstruation and pregnancy on HS.

**Methods:**

Two anonymous online surveys were created on Microsoft Forms and distributed via social media platforms (Facebook, Instagram, X and LinkedIn). The surveys were also shared via the HS Support Network UK and Ireland and HS Ireland’s mailing lists. The first survey explored the impact of menstruation on HS (open from 16 December 2024 to 18 January 2025); the second survey explored the impact of pregnancy on HS (open from 6 June to 20 August 2025). Survey data were analysed using descriptive statistics and free-text responses were coded, categorized into themes and analysed manually.

**Results:**

Of 255 participants who responded to the first survey, 96.1% (*n* = 245/255) noticed changes in their HS during their menstrual cycle. Of these, 99.6% (*n* = 244/245) experienced worsening symptoms. Of 43 respondents to the second survey, 58% (*n* = 25/43) reported HS worsening during pregnancy. Flare-ups typically occurred during the premenstrual phase and the second trimester of pregnancy. Respondents reported variable disease improvement with the antiandrogenic drugs metformin and spironolactone. Some respondents reported difficulty in caring for their newborn babies during the postpartum period, primarily because of pain caused by their HS.

**Conclusion:**

This study demonstrates a strong association between hormonal fluctuations and HS symptom deterioration. Clinicians should proactively assess menstrual patterns in HS management and provide support during the pregnancy and postpartum periods. Coordinated multidisciplinary care is essential. Further research is needed to evaluate antiandrogenic therapies for hormonally triggered HS exacerbations.

## Teaser

Hidradenitis suppurativa (HS) affects women three times more commonly than men, yet the influence of hormonal fluctuations on disease activity remains poorly quantified. Prior studies reported worsening of HS symptoms with menstruation in 43–77% of patients. This study explored the impact of menstruation, pregnancy and breastfeeding on HS, with information gathered from two anonymous surveys distributed via social media. The findings have practical implications for treatment and patient counselling.

What is already known about this topic?

- Hidradenitis suppurativa (HS) is three times more common in women than men in Europe and is associated with hormonal fluctuations during menstruation and pregnancy.
- Existing survey data report rates of menstrual worsening ranging from 43% to 77%, with inconsistent findings for pregnancy, reflecting significant heterogeneity across studies.
- Antiandrogen treatments, including spironolactone and metformin, are used in HS management, despite limited and underpowered evidence for efficacy.

What does this study add?

- A total of 96.1% of respondents reported menstrual cycle-related HS changes, with 99.6% describing worsening symptoms, predominantly in the premenstrual phase.
- Over 50% of respondents reported worsening of their HS during pregnancy, with symptom changes most commonly occurring in the second trimester.
- HS impacted on breastfeeding and the ability to care for a newborn baby, highlighting a postpartum burden.

Hidradenitis suppurativa (HS) is a painful chronic skin condition that causes abscesses, nodules and tunnelling in the skin folds. In Europe, it is three times more common in female individuals than in male individuals.^1^

The role of androgens in the pathogenesis of HS is unclear. No significant difference in serum androgen levels in patients with HS vs. control participants has been observed.^2^ It is thought that androgens may play a role by increasing the proliferation of follicular keratinocytes.^3^ Studies have reported disease worsening of HS symptoms (flare-ups) related to the menstrual cycle and pregnancy. Small interventional studies have reported the effectiveness of hormonal antiandrogens in the treatment of HS.^4^ This study aimed to explore the impact of hormonal changes during menstruation and pregnancy on the self-reported severity of HS symptoms.

## Materials and methods

### Surveys

Two anonymous online surveys were created with Microsoft Forms (Appendixes S1, S2; see Supporting Information). They were codeveloped with patient input and piloted with a patient with HS. The surveys were disseminated on social media platforms (Facebook, Instagram, X and LinkedIn), with the assistance of HS Support Network UK and Ireland and HS Ireland, with sharing enabled to promote distribution. The surveys were also shared via the organizations’ mailing lists. The first survey explored the impact of menstruation on HS (open from 16 December 2024 to 18 January 2025) and the second survey explored the impact of pregnancy on HS (open from 6 June 2025 to 20 August 2025).

### Statistical analysis

Survey data were analysed using descriptive statistics, with numerical data expressed as totals and percentages, where appropriate. Free-text responses were coded, categorized into themes and analysed manually using Microsoft Excel (version 16.105.3).

## Results

### Hidradenitis suppurativa and menstruation

Of the 255 individuals who responded to the first survey, 96.1% (*n* = 245/255) noticed changes in their HS during their menstrual cycle. Among those who reported changes, 99.6% (*n* = 244/245) experienced worsening symptoms, with only one respondent describing improvement of their HS during menstruation.

Most respondents (59.2%; *n* = 145/245) experienced premenstrual flares, while 30.2% (*n* = 74/245) reported menstrual flares. Only 5.7% (*n* = 14/245) experienced flares in the week following their period. The remaining respondents experienced changes throughout all 3 weeks or specifically around ovulation (Figure 1).

**Figure 1:**
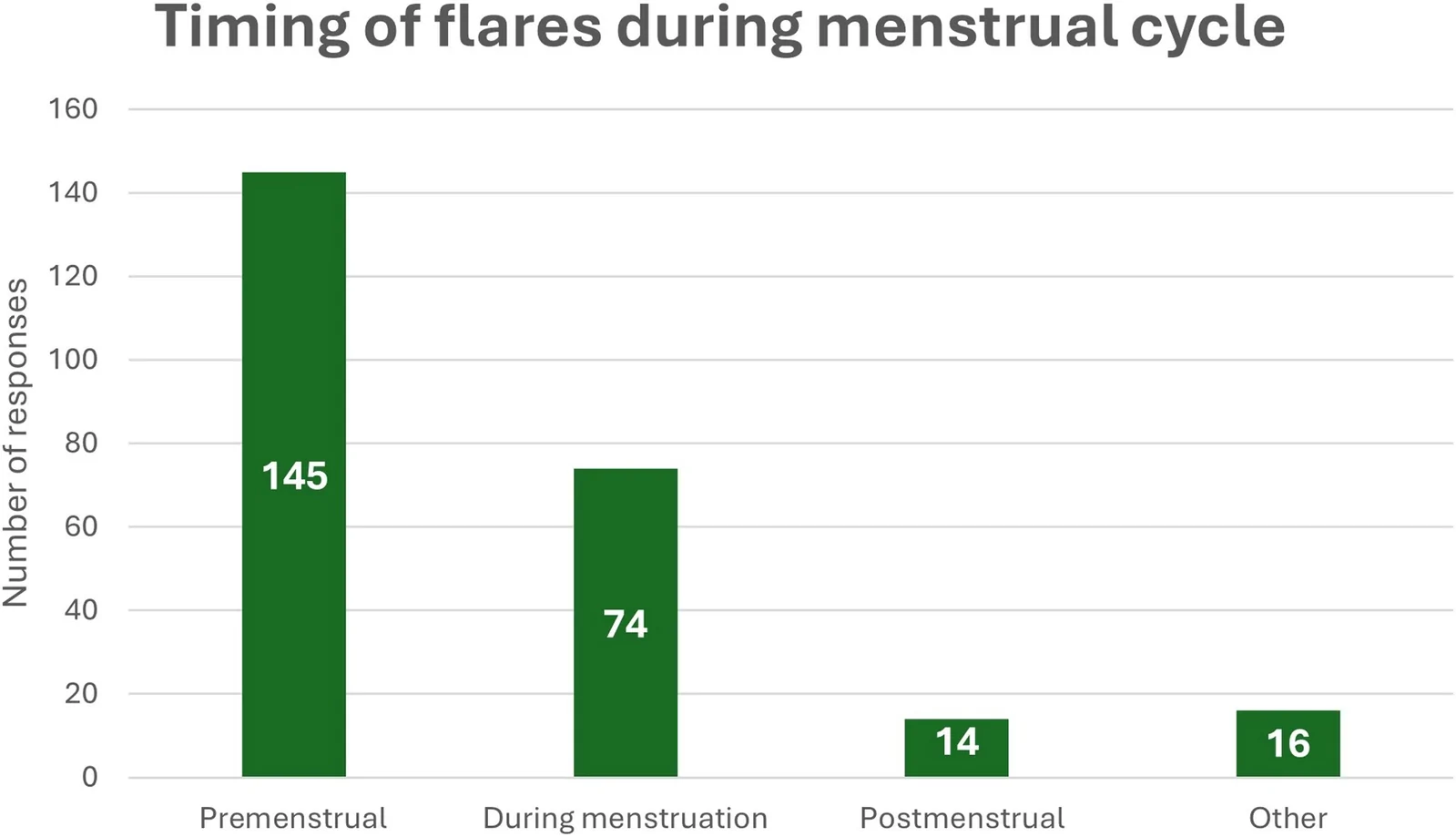
Timing of flares during the menstrual cycle.

### Hormonal treatments

Twenty per cent (*n* = 51/255) of respondents had been prescribed metformin, 9.8% (*n* = 25/255) spironolactone and 14.1% (*n* = 36/255) both medications. Of the 51 who had tried metformin only, 14 (27%) found it improved their HS. Additionally, of the 25 participants who had tried spironolactone only, 6 (24%) found it beneficial. Of patients who had tried either of these medications, 59.8% (*n* = 67/112) patients reported no improvement in HS symptoms. The free-text responses about these drugs were analysed and categorized into positive and negative comments: 33.3% of the free-text responses were positive and 66.6% were negative.

### Hidradenitis suppurativa and pregnancy

The second survey had 43 respondents, of whom 88% (*n* = 38/43) described a change in their HS during pregnancy. Most (58%; *n* = 25/43) reported HS worsening during pregnancy, while 30% (*n* = 13/43) experienced symptom improvement.

Among those who described changes in their HS during pregnancy, 50% (*n* = 19/38) reported changes during the second trimester, with smaller proportions reporting changes during the first (26%; *n* = 10/38) and third (11%; *n* =  4/38) trimesters. A small number (13%; *n* = 5/38) described changes throughout all three trimesters of pregnancy. Although no respondents reported postpartum flares for this question, there were two free-text comments regarding new flares along surgical scars following caesarean section (Figure 2).

**Figure 2:**
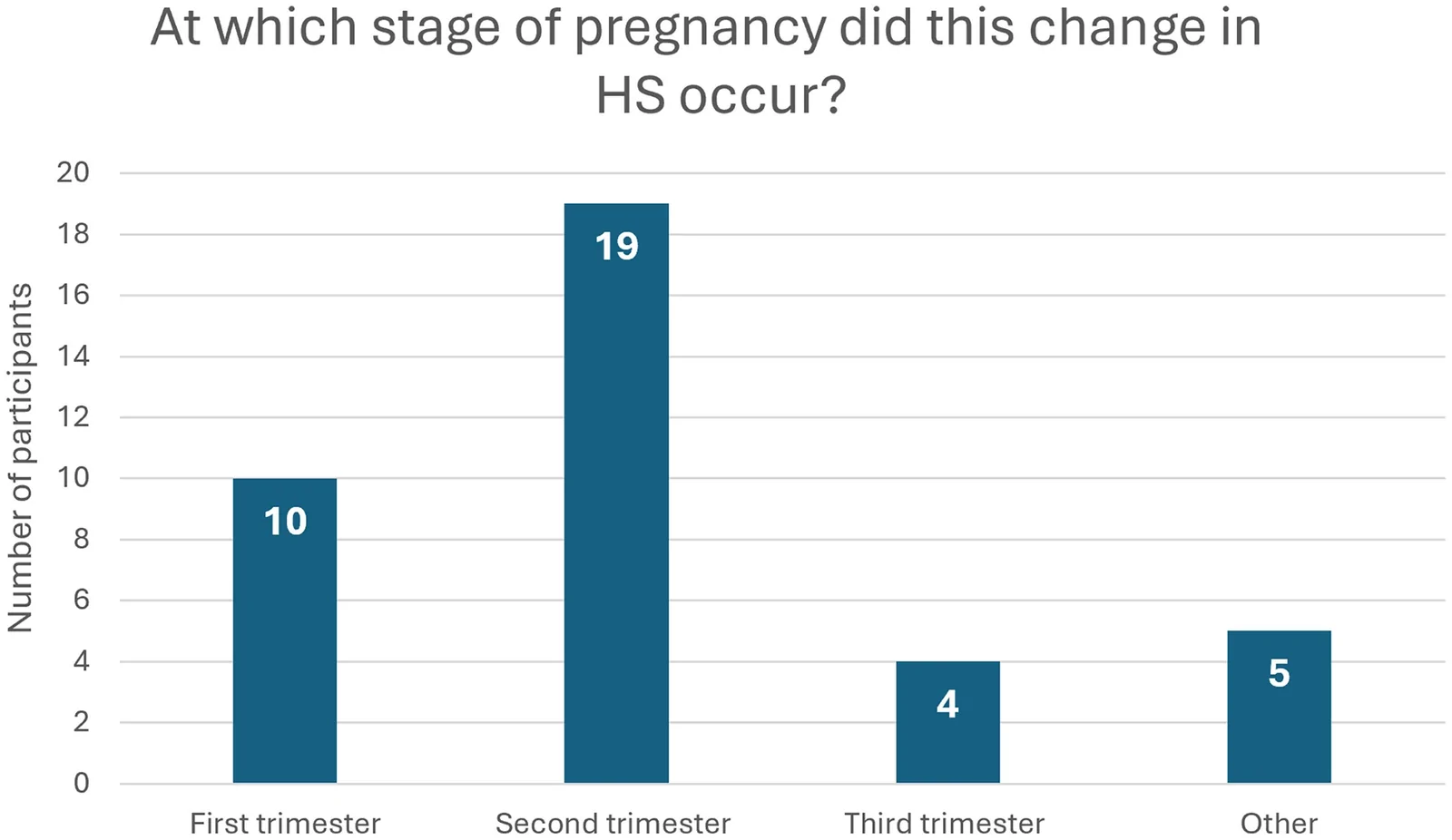
Timing of flares during pregnancy.

Of the 42% (*n* = 18/43) of respondents who had multiple pregnancies, 61% (*n* = 11/18) reported similar changes in their HS symptoms during each pregnancy, whereas 39% (*n* = 7/18) described no consistency in their HS symptoms across pregnancies.

Following pregnancy, 26% (*n* = 11/43) of respondents reported that their HS impacted on their ability to care for their newborn baby. The most frequently described challenge was pain secondary to HS, affecting their physical ability to hold, feed and care for their newborn baby, in addition to the mental and physical burden of HS.

In total, 42% (*n* = 18/43) of respondents breastfed their babies, of whom 39% (*n* = 7/18) reported that their HS affected breastfeeding. Free-text responses cited pain and discomfort as the primary reasons for this. One respondent reported that ‘[their HS] affected [their] confidence to feel comfortable breastfeeding, [their] skin was painful, and [they were] scared to pass on any bacteria or skin issues to [the] baby’, while another wrote, ‘breast milk leaked and flared my skin up’.

## Discussion

This study highlights the impact of hormonal fluctuations on HS severity in women of childbearing age, with most respondents reporting worsening symptoms during their menstrual cycle and pregnancy, with additional challenges during the postpartum period.

The limitations of this study include its small sample size, particularly for the second survey. Recruitment through social media increased the risk of selection bias as individuals who engage with HS support groups and online communities may have more severe disease or poorer treatment responses and are therefore more motivated to participate in research. Those with mild or well-controlled disease may therefore be underrepresented, which may have overinflated our findings. Further overinflation may have occurred due to response bias, as participants who experienced hormonal impacts on their HS were more motivated to participate. Self-reported disease activity during hormonal periods may reflect overlapping symptoms such as pain, fatigue and mood disturbance common at these times, rather than true changes in HS disease activity. Our results are based on self-reported survey data, particularly regarding disease severity, and remain observational rather than causal.

Future work could include prospective designs with larger sample sizes and clinician-assessed outcomes. Regarding generalizability, these results are most relevant to women of childbearing age who menstruate, with limited benefits for older patients, men and boys, and others with no childbearing potential.

On review of the existing literature, a 2019 survey distributed via social media showed that 76.7% of respondents experienced worsening of their HS with menstruation and 34.8% during pregnancy.^5^ Both results are lower than those found in our study, and they did not explore the changes over the course of pregnancy or the menstrual cycle. A 2017 survey reported menstrual deterioration of HS in 43.0% of participants, while findings for pregnancy contradicted ours, with 53.1% reporting no change in symptoms.^6^ A 2014 study supported this, finding that 72.0% of women reported no pregnancy-­related effects on their HS.^7^ Notably, one study that examined temporal changes found significant differences between trimesters, with the third trimester being most associated with symptom worsening (41.0%).^8^ These inconsistent findings across studies highlight the heterogeneous effects of menstruation and pregnancy on HS.

Despite the theoretical link between androgens and HS, there is limited evidence supporting the use of antiandrogens in treatment.^9^ An indirect connection exists through metabolic syndrome, a known disease association in HS, where increased insulin resistance stimulates androgen production. However, most patients with HS do not have hyperandrogenaemia, and it is postulated that end-organ hypersensitivity to androgens, rather than elevated circulating levels, drives the pathological process.^2,4^ Both spironolactone, a potassium-sparing diuretic with anti­androgen properties, and metformin, which indirectly affects androgens by reducing insulin resistance, have been explored as potential treatments. Spironolactone has shown some efficacy in treating HS, although studies have been underpowered.^10^ In our study, participants reported a lack of efficacy with spironolactone, although details on treatment duration or disease severity were not collected to contextualize these findings. Similarly, minimal benefits were observed with metformin treatment in our cohort. Further work is needed to explore the efficacy of antiandrogen therapies and their impact on disease progression associated with menstruation or pregnancy.

Sex hormones exert immunomodulatory effects that may be relevant to HS pathogenesis. Oestrogen has a proinflammatory effect through modulation of the innate and adaptive immune system, and enhances the severity of autoimmune conditions such as systemic lupus erythematosus.^11^ Oestrogen levels are highest in the follicular phase, peaking just before ovulation.^12^ In contrast, progesterone has an immunosuppressive effect, favouring a T helper 2-like cytokine profile^13^ and downregulating proinflammatory cytokines such as tumour necrosis factor-α and interferon-γ.^14^ This shift in immune balance is thought to facilitate successful fertilization and implantation following ovulation.^13^ Progesterone levels rise after ovulation, peaking 6–10 days later before rapidly declining in the absence of fertilization, triggering menstruation (Figure 3). This decline may explain the premenstrual flare-ups in over half of our respondents. However, conflicting evidence suggests that HS symptoms may remain stable or worsen during menopause, when oestrogen levels decline,^15^ indicating that the hormonal mechanisms underlying HS activity are not explained by oestrogen alone. Other factors, including prolactin, and nutritional and metabolic factors may contribute to HS severity.^15^

**Figure 3:**
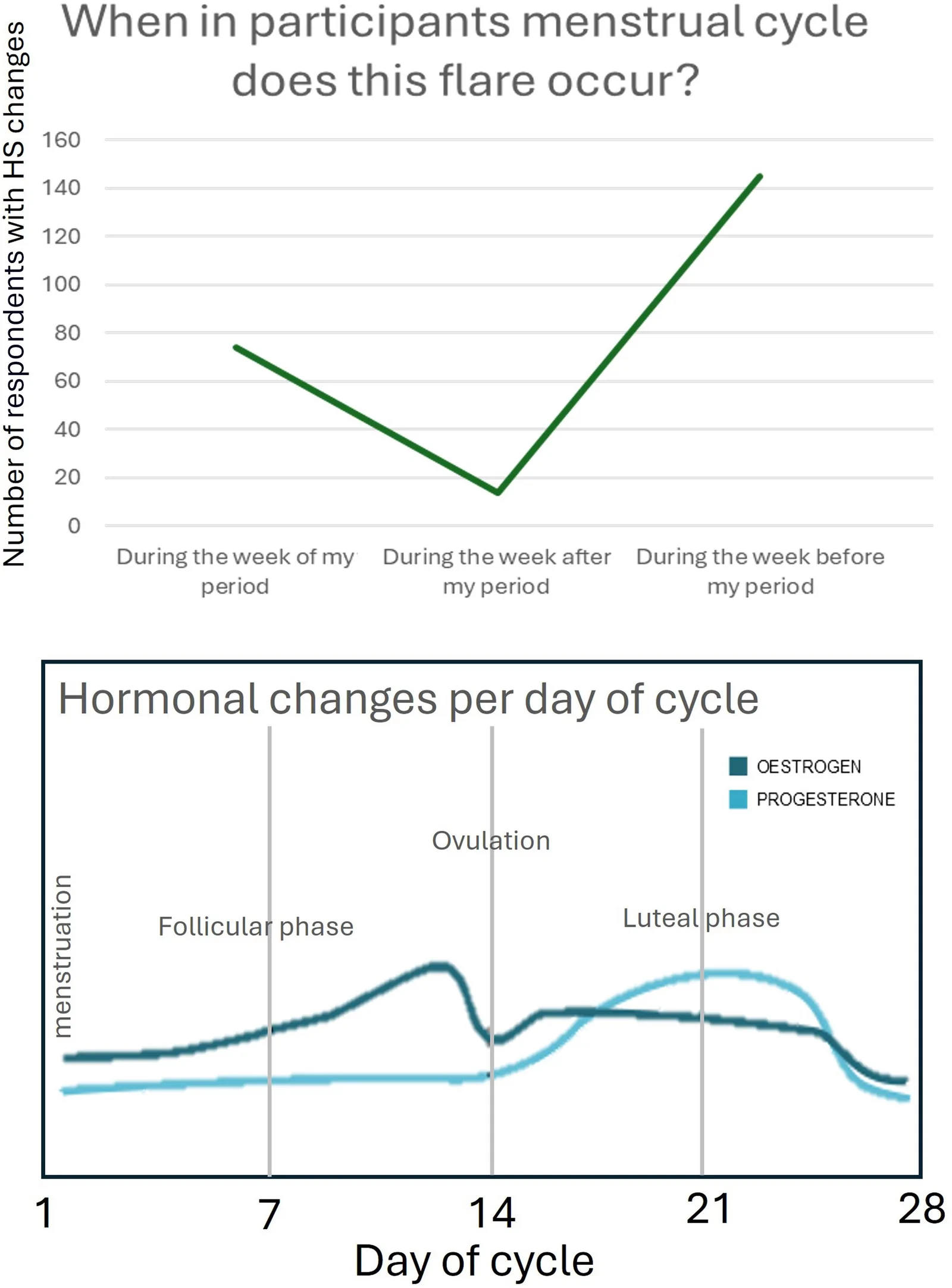
Flares and hormonal changes in the menstrual cycle. HS, hidradenitis suppurativa.

In conclusion, this study highlights the association between the deterioration of HS symptoms and both menstruation and ­pregnancy. It is important for clinicians to ask about disease fluctuations during menstruation and support patients with HS throughout pregnancy. The complex interplay between endocrine, reproductive and dermatological factors in women with HS underscores the value of coordinated care across dermatology, primary care, gynaecology, obstetrics and endocrinology.

## Supplementary Material

vzag087_Supplementary_Data

## Data Availability

The data underlying in this article are available in the article and in its online Supporting Information.
