# Supplementary material for: ‘My HS is significantly impacted by my cycle’: a cross-sectional study exploring the impact of menstruation and pregnancy on hidradenitis suppurativa
Source: Skin Health Dis. 2026 Jul 6;6(5):749–53. doi: 10.1093/skinhd/vzag087 (PMC13623445; doi:10.1093/skinhd/vzag087)
Supplement: Supplementary file 1 [file vzag087_supplementary_data.zip › Supplementary 2 HS and pregnancy questionnaire.pdf]

University of BRISTOL

HS and Pregnancy questionnaire

We are currently doing some research on whether people with hidradenitis suppurativa (HS) experience any changes in their HS when they are pregnant. If you are happy to take part, please fill out the survey below! The survey is 10 questions long and should take less than 15 minutes to complete.
Please see the Participant Information Sheet below for further information about the study:https://uob-my.sharepoint.com/:w:/g/personal/wk19475_bristol_ac_uk/Ee4bfuHJAVVHiGmdWPWUegIB6kc-g1zDdRVI3AGiJNC0vg?e=olqOYx

* This form will record your name, please fill your name.

1. Do your periods affect your HS?

- ◯ Yes, they worsen my HS
- ◯ Yes, they improve my HS
- ◯ No change to my HS

2. Have you been or are you currently pregnant?

- ◯ I am currently pregnant
- ◯ I have previously been pregnant
- ◯ I have never been pregnant (my participation ends here)

3. Has pregnancy affected your HS?

- ◯ Yes, it has improved my HS
- ◯ Yes, it has worsened my HS
- ◯ No, I have noticed no change in my HS with pregnancy
- ◯ Not applicable - I did not have HS when pregnant

4. If yes, when did this change occur?

- ◯ First trimester (weeks 1-12 of pregnancy)
- ◯ Second trimester (week 13-27 of pregnancy)
- ◯ Third trimester (week 28-40 of pregnancy)
- ◯ Postpartum (the first few weeks after having a baby)
- ◯ Other (please specify below)

5. If you selected other, please explain the change you experience below

6. If you have had multiple pregnancies, was this change similar in each pregancy or did it vary?

- ◯ My HS changes were similar with each of my pregnancies
- ◯ My HS changes varied with each pregnancy
- ◯ Not applicable - I have not had multiple pregnancies

7. If you breastfed your baby, did your HS affect this?

- ◯ Yes, my HS affected breastfeeding
- ◯ No, my HS did not affect breastfeeding
- ◯ I did not breastfeed

8. If your HS affected breastfeeding, please explain how below

9. Did your HS affect the ability for you to take care of your newborn baby?

- ◯ Yes
- ◯ No
- ◯ Unsure

10. If your HS affected the ability for you to care for your newborn, please explain how below

11. Space to add any additional comments (optional)

This content is neither created nor endorsed by Microsoft. The data you submit will be sent to the form owner.

Microsoft Forms
