# Supplementary material for: ‘My HS is significantly impacted by my cycle’: a cross-sectional study exploring the impact of menstruation and pregnancy on hidradenitis suppurativa
Source: Skin Health Dis. 2026 Jul 6;6(5):749–53. doi: 10.1093/skinhd/vzag087 (PMC13623445; doi:10.1093/skinhd/vzag087)
Supplement: Supplementary file 1 [file vzag087_supplementary_data.zip › Supplementaryá1 HS and periods questionnaireá.pdf]

University of BRISTOL

HS and Periods Questionnaire

We are currently doing some research on whether people with hidradenitis suppurativa experience any changes in their HS when they have their periods. If you are happy to take part, please fill out the survey below! The survey is 7 questions long (5 multiple choice questions and 2 written answer question) and should take less than 10 minutes to complete.

More information for participants: *Hidradenitis suppurativa (HS) is a painful, chronic skin condition that causes skin abscesses and scarring. It commonly occurs near hair follicles where there are sweat glands, usually around the groin, bottom, breasts and armpits.*

The study we are completing is called 'a scoping review of the impact of the menstrual cycle on exacerbations of hidradenitis suppurativa' and will be about how participants menstrual cycles affect their hidradenitis suppurativa.

Taking part in this research will involve filling in this short survey about whether your period affects your HS. The answers will then be used in the study, and any written responses may be included too. Participants identifiable information (e.g. names, email addresses) will not be stored or shared. Written responses will be deidentified.
Participation in this study is not mandatory, you are free to withdraw your research data at any time before submission by closing the browser, without a reason. Consent in implied by clicking on and completing this survey. Once submitted you will have consented to participate and data cannot be withdrawn.

This survey is being shared by HS Ireland and the HS Network social media portals and anyone on these social media platforms with HS who menstruates can take part. The data will be stored on secure University of Bristol servers. At the end of the study the results will be anonymised and quotations may be included in publications. The research data will then be deleted. The results of this project may be published and presented at HS conferences.

The benefits of taking part in this study are that results could be used in future studies to improve the management of HS. The disadvantages of taking part are that there is no guarantee the results may benefit future treatment.

This study has been peer reviewed by Dr Hannah Wainman and has received faculty ethics approval (HSSREC : 22244).
If you have any questions or concerns before deciding to take part in this study please contact the study lead Olivia Garrett: wk19475@bristol.ac.uk.
All of the above information can be saved if you save your response to the survey.

Thank you for your time and participation!

1. **Do your periods affect your HS?**

- ◯ Yes
- ◯ No

2. **If yes, how do they affect your HS?**

- ◯ My HS improves
- ◯ My HS worsens
- ◯ N/A or no change

3. **When does this change occur?** (if applicable)

- ◯ During the week before my period
- ◯ During the week of my period
- ◯ During the week after my period
- ◯ Other

4. **Have you ever taken spironolactone or metformin for your HS?**

- ◯ Yes, spirinolactone
- ◯ Yes, metformin
- ◯ Yes, both
- ◯ No

5. **If you tried either of these drugs, what impact did they have on your HS (if any)?** Please note any written responses given here may be anonymised and included in publications

6. **Space to give any more information you wish** (optional) Please note any written responses given here may be anonymised and included in publications

This content is neither created nor endorsed by Microsoft. The data you submit will be sent to the form owner.

Microsoft Forms
